# Mechanism of Assembly of the Non-Covalent Spectrin Tetramerization Domain from Intrinsically Disordered Partners

**DOI:** 10.1016/j.jmb.2013.08.027

**Published:** 2013-09-17

**Authors:** Stephanie A. Hill, Lee Gyan Kwa, Sarah L. Shammas, Jennifer C. Lee, Jane Clarke

**Affiliations:** 1 - University of Cambridge Chemical Laboratory, Lensfield Road, Cambridge CB2 1EW, UK; 2 - Laboratory of Molecular Biophysics, Biochemistry and Biophysics Center, National Heart, Lung, and Blood Institute, National Institutes of Health, Bethesda, MD 20892, USA

**Keywords:** protein folding, protein engineering, Φ-value analysis, IDP, natively unfolded protein

## Abstract

Interdomain interactions of spectrin are critical for maintenance of the erythrocyte cytoskeleton. In particular, “head-to-head” dimerization occurs when the intrinsically disordered C-terminal tail of β-spectrin binds the N-terminal tail of α-spectrin, folding to form the “spectrin tetramer domain”. This non-covalent three-helix bundle domain is homologous in structure and sequence to previously studied spectrin domains. We find that this tetramer domain is surprisingly kinetically stable. Using a protein engineering Φ-value analysis to probe the mechanism of formation of this tetramer domain, we infer that the domain folds by the docking of the intrinsically disordered β-spectrin tail onto the more structured α-spectrin tail.

## Graphical Abstract

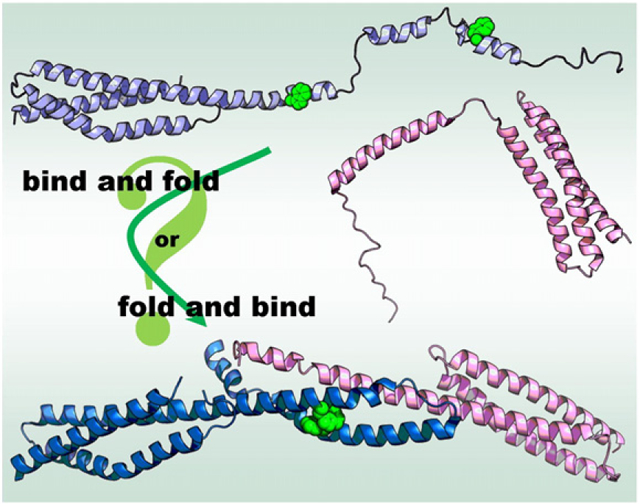

**Legend:** Protein engineering methods may be used to investigate the mechanism of complex formation from disordered partners, discriminating binding-and-folding from folding-and-binding mechanisms. Simulated structure of human erythroid β-spectrin C-terminal domains previously published as Shammas, S. L., Rogers, J. M., Hill, S. A., & Clarke, J. (2012). Slow, reversible, coupled folding and binding of the spectrin tetramerization domain. Biophys J 103, 2203–14. N-terminal domains of human erythroid α-spectrin PDB ID: 1OWA; erythroid spectrin tetramer domain PDB ID: 3LBX. Structures prepared in PyMol (DeLano Scientific).

## Introduction

Spectrin is an elongated structural protein common to all animal species and requisite for life [1,2]. Although non-structural functions of alternative isoforms in other tissues have now been reported [3–7], spectrin was originally known for its structural support properties in erythrocytes [8], where it forms extensive two-dimensional networks through interactions with itself and other proteins while binding elements of the three-dimensional cytoskeleton, such as ankyrin and adducin [2,9–11].

**Fig. 1.**
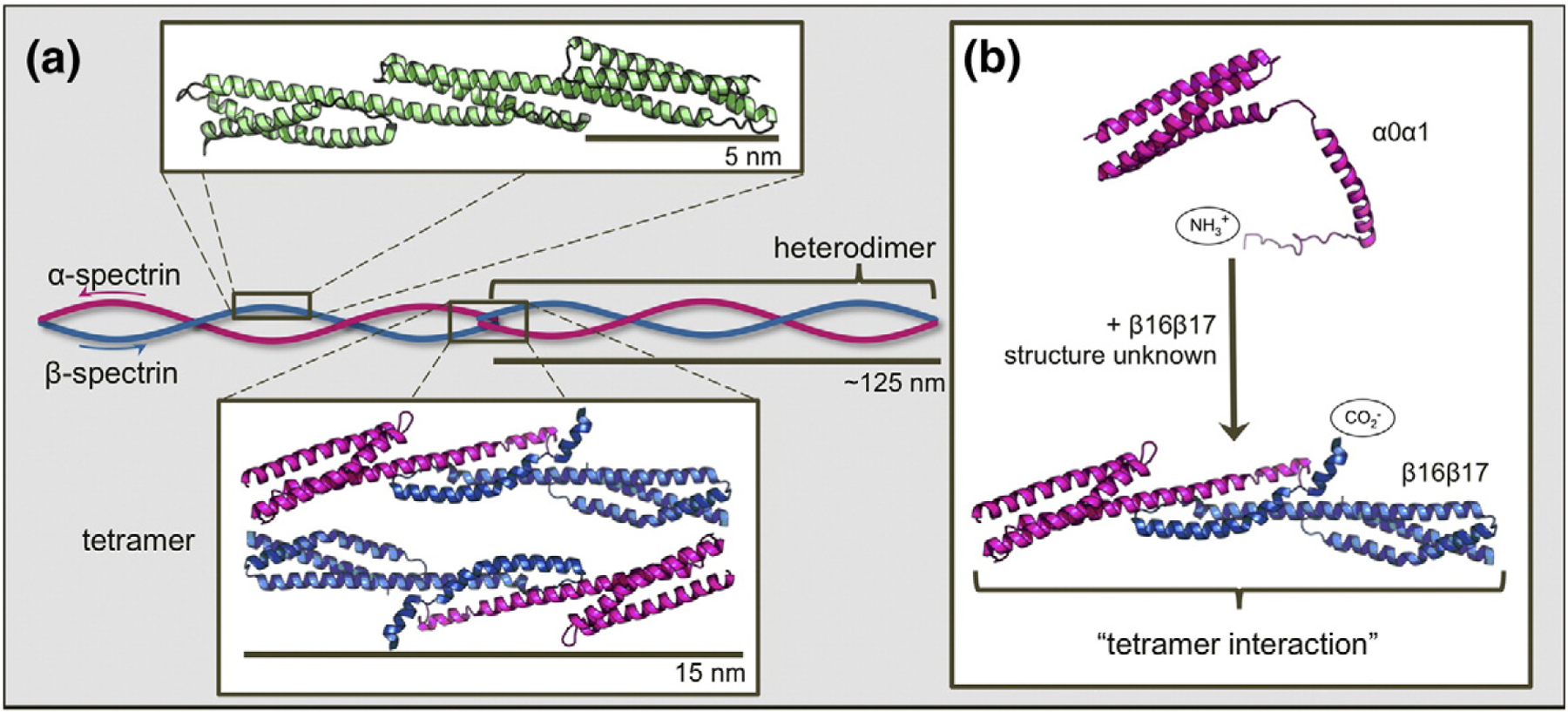
Schematic depiction of the structure and assembly of erythrocyte spectrin. (a) Three contiguous spectrin domains (green, chicken brain α-spectrin domains R15, R16, and R17; PDB ID: 1U4Q) show the three-helix-bundle structure characteristic of tandem spectrin domains. A continuous helix connects the C-helix of one domain to the A-helix of the following domain. Subunits α-spectrin and β-spectrin (pink and blue, respectively) associate laterally to form heterodimers. Where two heterodimers meet “head on”, the C-terminal tail of β-spectrin binds and folds with the N-terminal tail of α-spectrin of the opposing heterodimer to form a new, non-covalent three-helix bundle. This is termed the “tetramerization domain” (PDB ID: 3LBX). (b) Truncated constructs α0α1 (PDB ID: 1OWA) and β16β17 are employed to facilitate study of the tetramer domain as a dimerization process. Structures prepared using PyMOL (DeLano Scientific).

Spectrin networks have many strata of structure, described in Fig. 1. The basic unit of structure is the “spectrin domain”, a 106-residue, 5-nm-long three-helix bundle, with helices designated A, B, and C [12,13]. Spectrin domains are arranged as tandem repeating units with significant sequence homology, where Helix C of one domain is contiguous with Helix A of the next domain [14]. These tandem arrays of spectrin domains form two homologous multidomain proteins, subunits called α-spectrin and β-spectrin. The two subunits found in erythrocyte spectrin are of different lengths and compositions: α-spectrin contains 20 full spectrin domains, the first of which is preceded by a partially helical N-terminal tail, while β-spectrin has only 16 full spectrin domains, followed by a C-terminal tail. β-Spectrin also has two additional CH domains at the N-terminus that bind actin, protein 4.1, adducin, and PIP_2_ and function to assemble the spectrin network onto the erythrocyte membrane [2]. Single α- and β-subunits associate laterally to form an antiparallel heterodimer, approximately 125 nm in length when extended and visible by electron microscopy [15]. Finally, two heterodimers associate laterally in what is termed a “head-to-head” manner to form a spectrin tetramer—the functional unit. Our study investigates the mechanism of formation of the interactions at this tetramer junction.

Two head-to-head interactions between the terminal tails of two heterodimers form the final stratum of structure, the erythrocyte spectrin tetramer. In each interaction, the N-terminal tail of α-spectrin binds the C-terminal tail of β-spectrin from the other heterodimer, forming a new three-helix spectrin-like domain (Fig. 1). This non-covalent three-helix bundle is commonly known as the “spectrin tetramer domain” (even though it is, in fact, a heterodimer between just one α-spectrin and one β-spectrin molecule) [16]. In the study of formation of this tetramer domain, truncated constructs that eliminate the possibility of heterodimer formation by removing the domains responsible for lateral interaction are frequently employed [17,18]. Since the adjacent complete spectrin domains, α1 and β16, are not involved in heterodimer formation [17,19,20], we use a truncated form of α-spectrin that contains only the N-terminal tail and first full domain in α1 (α0α1) and the final full domain in β-spectrin with the C-terminal tail (β16β17). Thus, we can study assembly of the tetramer domain in the absence of lateral interactions between heterodimers (Fig. 1b) [21,22]. We note that previous attempts to study this interaction using the unstructured tails alone have proved unsuccessful [23].

Two structures relevant to the erythrocyte spectrin tetramer exist: an NMR structure of α0α1 [24] (PDB ID: 1OWA) and a crystal structure of the tetramer site [25] (PDB ID: 3LBX), which shows that the tails of each subunit fold together to form a new spectrin-like domain [26], as previously predicted from homology studies [27]. Comparing the structures of α0α1 in isolation to the tetramer interaction, it is evident that the only significant structural change in α0α1 is the formation of a linking helical region that joins Helix A in the first full domain to the helix in the tail region (Helix C of the tetramer) [28]. In contrast to the abundance of information regarding α0α1, little is known about the structure of β16β17 in isolation. Circular dichroism (CD) has shown a significant increase in helicity upon formation of the tetramer interaction [22]; the majority of this increase can be attributed to increase in helical content of the β17 tail, as apparently only four additional residues become helical in α0α1 upon tetramer formation. It is generally inferred that the C-terminal tail of β16β17 is relatively unstructured in isolation.

Thus, the β17 tail has characteristics of an intrinsically disordered protein (IDP). IDPs feature an unstructured region that, in some cases, binds to structured proteins, adopting a folded structure (often α-helical) in the process [29–31]. Mechanisms for IDPs associating with their partner protein are currently an area of significant interest, for which two extreme possible mechanisms have been proposed [32,33]. First, IDPs can bind their partner *via* a conformational selection mechanism, where the IDP is fully folded before binding [34]. Alternatively, IDPs can bind to their partner in a fully disordered form and then fold using the partner protein as a scaffold [35]. Although not an example of an archetypal IDP, study of the mechanism of spectrin tetramer interaction may provide insights into IDP folding mechanisms more generally.

Interest in the spectrin tetramer has been driven by the identification of mutations in the spectrin tetramer region linked to hereditary elliptocytosis and spherocytosis, which are anemias characterized by anomalous erythrocyte morphology [36,37]. Previous investigations of the tetramer interaction have mainly used structural [38] and equilibrium [39] techniques, as well as molecular dynamics simulations [40]. Isothermal titration calorimetry (ITC) of the system has been performed by many groups, revealing a dissociation constant (*K*_d_) of about 0.4 μM for wild type. ITC has also been performed on a small subset of mutants [41,42]. Two studies have investigated the kinetics of tetramer formation. A study utilizing surface plasmon resonance has reported the rate constant for association of the spectrin tetramer interaction (*k*_+_) to be 60 M^−1^ s^−1^ [43]. More recently, our laboratory has developed a (solution-based) stopped-flow approach based on intrinsic tryptophan fluorescence, capable of extracting the association and dissociation rate constants for spectrin tetramerization [44]. We found the association rate constant (630 M^−1^ s^−1^) to be an order of magnitude larger than that reported from the surface-based approach.

Protein engineering methods, particularly Φ-value analysis, have played an invaluable role in elucidating folding mechanisms for a variety of proteins [45,46]. Of special relevance to this study are the previously published Φ-value analyses of three individual spectrin domains from chicken brain α-spectrin. These domains, called R15, R16, and R17, have high sequence and structural homology but fold at dramatically different rates. Through extensive mutation, it has been shown that R15, the fastest folder, features a strong nucleus during folding, while R16 and R17 lack such a nucleus and fold primarily through the docking of preformed elements of secondary structure [47–50]. Regardless of folding mechanism, the order of helix folding for each studied domain has proved to be the same: Helices A and C dock and fold first, followed by the docking of Helix B.

Here, we extend the Φ-value analysis technique to study the mechanism of folding/assembly of the tetramer domain of spectrin.

## Results

### Design of the constructs

Four constructs were designed for this study: β16, β16β17, α1, and α0α1. Domain boundary selection for the two β-spectrin constructs was guided by previous work by Nicolas *et al*. [23]; β16 (residues 1898–2004) comprises the final full spectrin repeat in β-spectrin and β16β17 (residues 1898–2083) is β16 plus the incomplete 17th domain, which has homology for one A- and one B-helix. The published NMR structure for the N-terminal end of α-spectrin provided the domain boundaries for the first full spectrin domain, identifying this domain as S52-R156 [24]. However, since spectrin domains are defined as having 106 residues [51], we infer that the true domain C-terminal boundary is L158. In addition, MacDonald and Pozharski have shown that additional (native) residues at both N- and C-terminal ends of single spectrin domains significantly improve domain stability [51]. Accordingly, we add four residues to the beginning of the domain, and five to the end, yielding our α1 construct as residues 48–163. The helix in the first (incomplete) domain of α-spectrin begins at residue 19; however, a construct containing residues 19–163 (α0α1′) does not bind β16β17 to yield the tetramer interaction (data not shown). Given this, we include much of the unstructured N-terminal tail, selecting residues 2–163 for construct α0α1.

Two residue identification systems are in common use to describe spectrin: R15, R16, and R17 are numbered individually as domains, while the human spectrin proteins are typically numbered in reference to the entire protein. With the use of domain-wise numbering, the tetramer covers residues 1–108, with residues 1–77 on β16β17 and residues 79–108 on α0α1. Alternatively, with the use of protein-specific numbering, the β16β17 contribution to the tetramer is residues 2004–2079; α0α1 contributes residues 19–49. Unless otherwise noted, the domain-wise numbering scheme is used throughout the text, in order to facilitate comparison with previous Φ-value analyses of spectrin.

### Wild-type spectrin

**Fig. 2.**
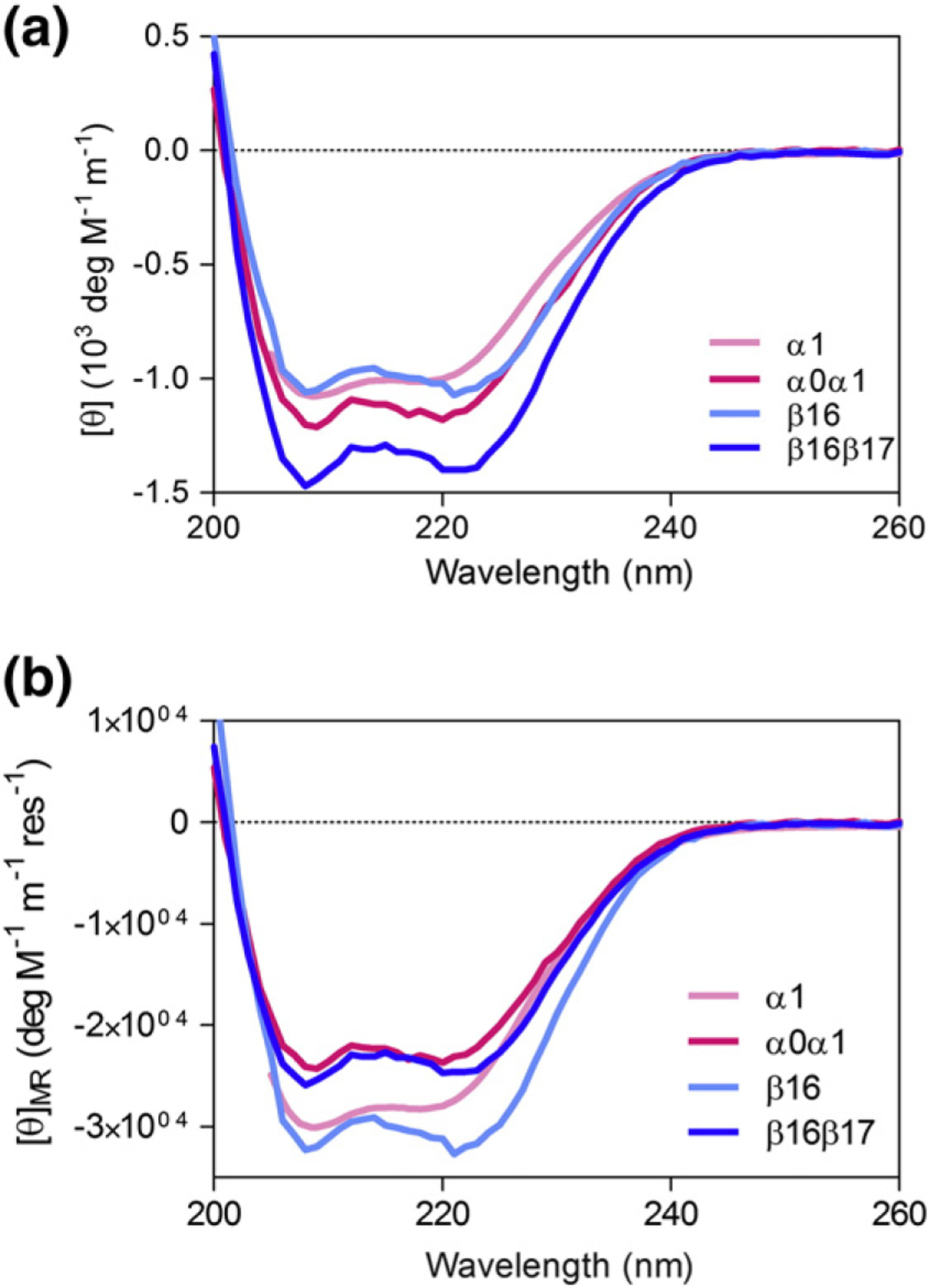
CD profiles for erythrocyte spectrin constructs. (a) Equal concentrations of α1 and β16 have similar CD spectra, as we might expect from 2 three-helix bundles of similar size. Addition of α0 to α1 and β17 to β16 shows an increase in overall helicity, suggesting that both α0 and β17 have some helical structure. (b) However, when we compare mean residue ellipticity (MRE), it is clear that the MRE values for α0α01 and β16β17 are significantly lower in magnitude than those for folded α1 and β16, respectively. Both tail regions are also significantly unstructured.

CD spectra of the extended constructs α0α1 and β16β17 were compared to those of the folded domains α1 and β16 (Fig. 2). From the data, it is clear that all four constructs are predominantly α-helical. Addition of the N-terminal tail to α1 increases helical content, as one would expect, given the NMR structure of α0α1, which shows an isolated helix from residue 19 to residue 46 (protein-specific numbering). Further, there may be some helix fraying in Helix A in α1, which may be alleviated by the addition of residues at the domain’s N-terminal boundary. With the use of the signal at 222 nm as a guide, the increase in helicity seen in α0α1 does not appear great enough to indicate complete formation of helix in the α0 domain despite α0 looking fully structured by NMR techniques [24]. Similarly, β16β17 has more helix than β16, perhaps by around as much as a single helix. This implies some degree of native helix in the β17 domain, which is as of yet unstudied by NMR. Our results cannot identify the location of this helicity, and it may be residual and spread across the tail. This would be consistent with results of equilibrium unfolding curves performed by CD, which showed no cooperative unfolding of α0 and β17 regions (data not shown). Although we did not include extra residues on the C-terminal end of β16, it is unlikely that the C-helix in the β16 construct is significantly frayed, given the results of equilibrium and kinetic experiments, as discussed below.

**Fig. 3.**
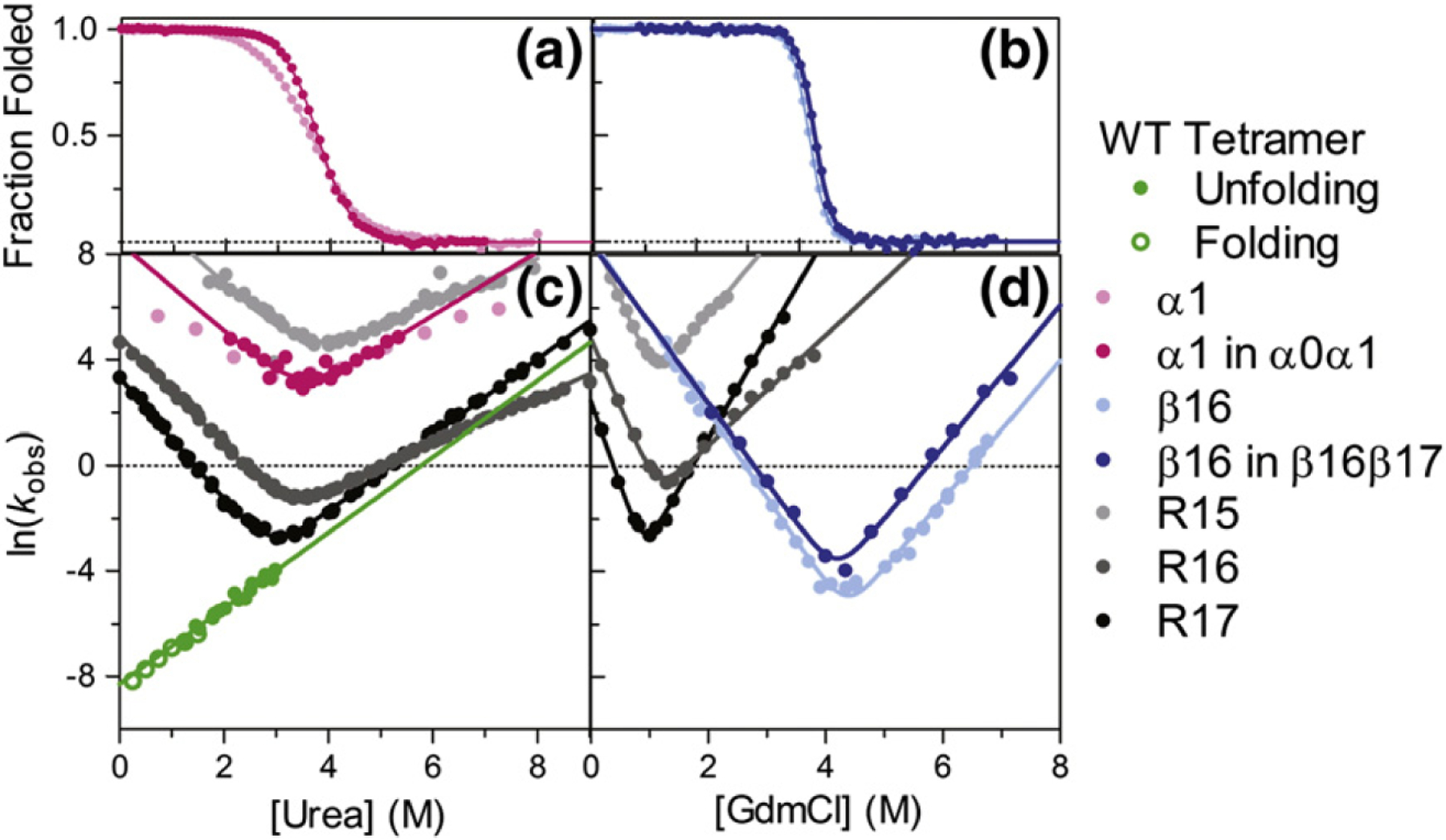
Characterization of α1, α1 in α0α1, β16, β16 in β16β17, and the tetramer domain in relation to R15, R16, and R17. (a) α1 in α0α1 and α1 alone have similar [Den]_50%_ values, but different *m*_D–N_. This probably reflects helix fraying in α1, in the absence of the N-terminal extension. (b) Addition of β17 does not alter the high stability of β16, which unfolds only in the presence of high concentrations of GdmCl. (c) Chevron plots for α1 and α1 in α0α1 are compared to R15, R16, and R17. Both α-spectrin constructs fold fast, but the decrease in *m*_kin_ in α1 is obvious in comparison to other spectrin domains (see the text). The observed rate constants for tetramer dissociation (green) are calculated from folding (open) and unfolding (closed) experiments and display a denaturant dependence (*m*_*k*−_), which is consistent with the ${\text{m}}_{k_{u}}$ for R17. Interestingly, the tetramer domain is much more kinetically stable than one of its neighboring domains, α1. (d) Chevrons for the β-spectrin constructs show that β16 alone and in β16β17 folds as quickly as R15, but due to its inordinate stability, it has very low unfolding rate constants.

The monomeric folding and unfolding of the wild-type constructs were studied by chemical denaturation experiments, utilizing intrinsic tryptophan fluorescence to monitor folding. Equilibrium results for the constructs indicate that both fold reversibly in a two-state manner (Fig. 3 and Table 1). We have previously observed that spectrin domains may be stabilized by unfolded neighboring domains [52]. Interestingly, α1 is stabilized by the addition of the α0 tail. The *m*-value of α0 alone is also lower than that of α0α1 and other previously studied spectrin domains (Table 1). This suggests that our α1 construct is cut “too short” in the construct and, consequently, the A-helix is frayed. Notably, α1 in the α1α0 construct has similar stability to R15, R16, and R17 [48,51]; however, β16 is extremely stable, with a free energy of unfolding (Δ*G*_D–N_) of 14.2 kcal mol^−1^ and a midpoint of >4 M guanidinium chloride (GdmCl). This stability is essentially unaffected by addition of the β17 tail. (Note that the equilibrium *m*-value of β16 is similar to that of R15, R16, and R17 in GdmCl.) Folding and unfolding kinetic data were well fitted by single-exponential functions. Plotting the natural logarithm of the observed rate constant, *k*_obs_, against denaturant concentration yields typical chevron plots; the refolding and unfolding limbs for both α1 and β16 are linear within the experimentally accessible range of denaturant. It can be seen that both fold as rapidly as R15; the stability of β16 arises from the decreased unfolding rate constant.

### Φ-Value analysis: 1. Choice of mutations

The selection of residues to mutate was guided by the residues studied in the Φ-value analyses of R15, R16, and R17 [47,49,50] and the crystal structure of the tetramer interaction. Mutants were created in each of the three helices of the tetramer domain, at both core and solvent-exposed residues. Mutations of buried residues were made to delete interactions in the core. Each residue was mutated to Ala, except native Ala residues, which were mutated to Gly. Additionally, aromatic residues were mutated to Leu. Surface residues were mutated to both Ala and Gly in order to investigate the extent of secondary structure formation at the transition state. In all, 23 mutations were made to probe 14 sites in α0α1, and 40 mutations were made to probe 27 sites in β16β17. CD spectra were collected for each mutant to assess the effect of the mutations on secondary structure of the monomer. Only L91A, Y94A, and L108A significantly reduced the helicity of α0α1 (Fig. S1); Φ-values were not calculated for either L91A or L108A since L91A had an unusual *m*-value and L108A abolished formation of the tetramer domain. Y94A yielded a non-classical Φ-value (see Discussion).

### Φ-Value analysis: 2. Collection of data

Mutant proteins were exclusively studied binding to their wild-type partner, never pair-wise with a mutant partner. Equilibrium data were collected by ITC, following the heat released as α0α1 binds β16β17 to form the tetramer domain. Fitting of these data yields association constants (*K*_a_ values), where $K_{\text{a}}=\frac{1}{K_{\text{d}}}$ [44]. Eight mutations effectively abolished binding (Tables 2 and 3); the *K*_d_ for wild type was 0.4 μM, and *K*_d_ values for mutant tetramer domains ranged from 0.15 to 170 μM. Changes in free energy of dissociation upon mutation, ΔΔ*G*_D–N_, are reported in Tables 2 and 3, with sample ITC traces in Fig. S2.

Kinetic data were collected with stopped-flow experiments, following the increase of intrinsic Trp fluorescence observed upon dissociation/unfolding of the preformed tetramer domain in the presence of urea.

### Φ-Value analysis: 3. Analysis of data

Although one might expect dissociation to be well represented by single-exponential kinetics, we have demonstrated previously that a second-order reversible model is needed to accurately represent the disassociation data despite the relatively weak association between α0α1 and β16β17 (*K*_a_ = 0.4 μM). All data were well fitted by this model [44].

$$F=F_{0}+\Delta F\times \frac{b-z-(b+z)\left(\frac{2{\left[AB\right]}_{0}+b-z}{2{\left[AB\right]}_{0}+b+z}\right)\text{exp}\left(zk_{+}t\right)}{2\left(\left(\frac{2{\left[AB\right]}_{0}+b-z}{2{\left[AB\right]}_{0}+b+z}\right)\text{exp}\left(zk_{+}t\right)-1\right)}$$

where *b* = −*K*_d_ − 2[*A*]_*t*_ and $z=\sqrt{K_{\text{d}}^{2}+4K_{\text{d}}{\left[A\right]}_{t}}$. *F*_*0*_ is the fluorescence at time zero, *F* is the fluorescence at time *t*, and *k*_+_ is the association rate constant. [*AB*]_0_, the concentration of dimer in the reaction mixture at time zero, is calculated from the *K*_d_ obtained from ITC experiments and the initial concentration of α0α1 ([*A*]_t_) in the protein solution prior to mixing with denaturant.


$$2{\left[AB\right]}_{0}=\frac{2{\left[A\right]}_{t}+K_{\text{d}}-\sqrt{{\left(K_{\text{d}}+2{\left[A\right]}_{t}\right)}^{2}-4{\left[A\right]}_{t}^{2}}}{2}$$

**Fig. 4.**
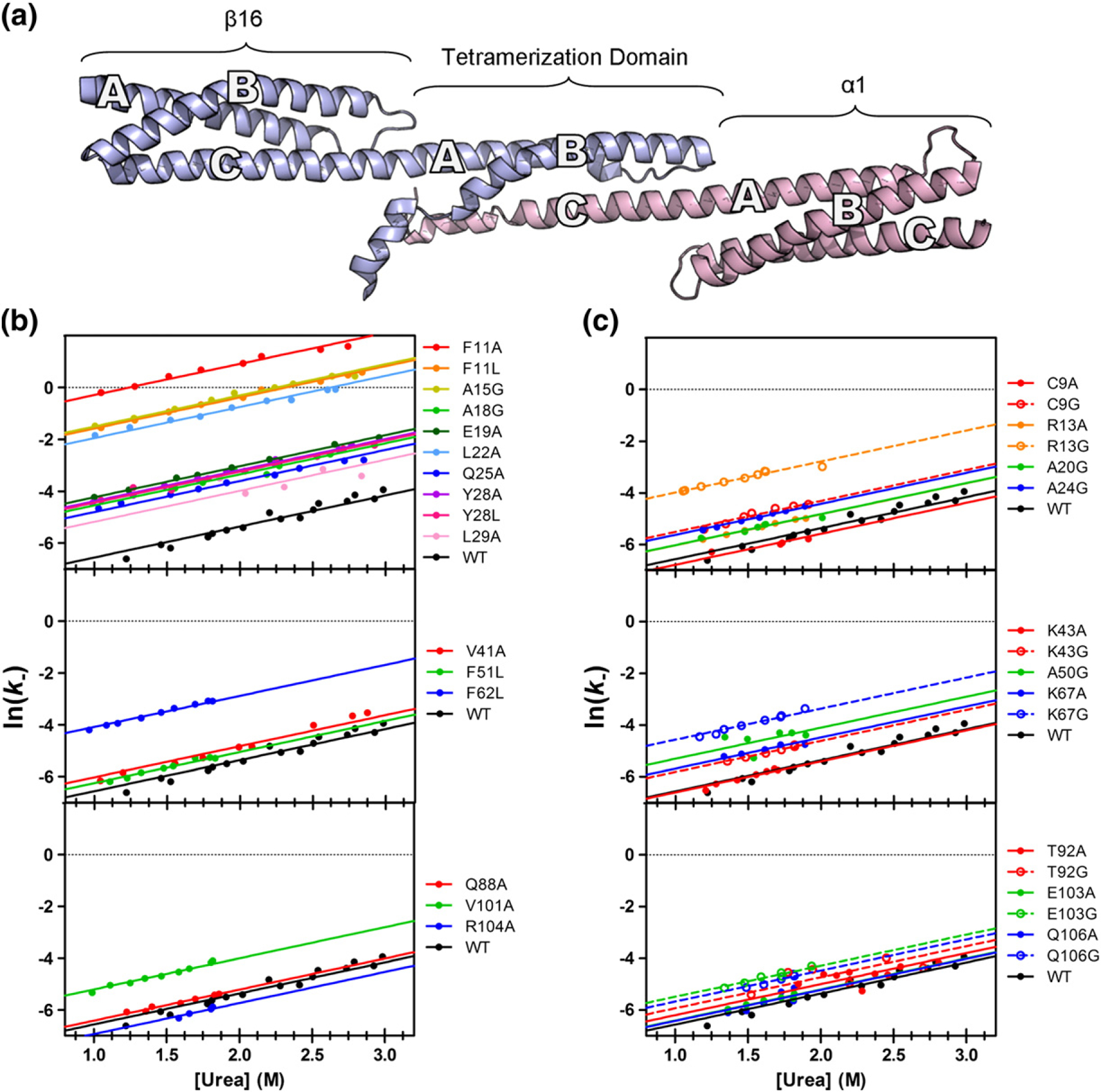
Structure of the tetramer domain and dissociation/unfolding kinetics for mutants. (a) β16β17, shown in blue, consists of a full three-helix domain (β16) followed by a tail (β17), forming the A- and B-helices of the tetramer domain. α0α1 (pink) has a full domain (α1), preceded by the N-terminal tail (α0), which forms the C-helix of the tetramer domain. (b and c) The observed rates for dissociation/unfolding experiments, used to calculate Φ-values for core and surface mutants, respectively. Mutants are divided into three groups: those found on Helix A (top), Helix B (middle), and Helix C (bottom). Each unfolding “limb” is well fitted with an *m*_*k*−_ of 1.2 kcal M^−1^. Mutations to the core of the A-helix show the greatest variation in observed dissociation/folding rate constants.

Dissociation rate constants for each mutant were plotted as a natural logarithm against the final urea concentration, yielding linear unfolding limbs for each mutant (Fig. 4). With only two exceptions, the unfolding limbs had very similar slopes; thus, these were fitted globally, yielding a shared *m*-value for disassociation, *m*_*k*−_, of 1.20 ± 0.02 M^−1^. This is similar to estimates of *m*_*k*−_ from association and dissociation studies of the wild type reported previously of 1.25 ±0.07 M^−1^ and 1.44 ± 0.07 M^−1^, respectively [44]. The *y*-intercept of each fit represents the natural logarithm of the unfolding rate in the absence of denaturant, $\text{ln}\left(k_{-}^{{\text{H}}_{2}\text{O}}\right)$.

From these unfolding data, it is possible to calculate Φ following the method of Fersht *et al*. [53].


$$\Phi =1-\frac{-RT\;\text{ln}\left(\frac{k_{-}^{\text{WT},{\text{H}}_{2}\text{O}}}{k_{-}^{\text{mut},{\text{H}}_{2}\text{O}}}\right)}{\Delta \Delta G_{\text{D}-\text{N}}}$$

The calculated Φ-values and dissociation rate constants (*k*_−_) are shown for the core and surface mutations in Tables 2 and 3, respectively.

## Discussion

### Characterization of spectrin domains: α0α1, β16β17, and the tetramer domain

In this work, we have characterized three new spectrin domains: α1, β16, and the tetramer domain. Of these, α1 behaves most similarly to the previously described R15, R16, and R17. As shown in Table 1, the thermodynamic stability of α1 (4.3 kcal mol^−1^ or 6.5 kcal mol^−1^ when extended in α0α1) fits well with the range of Δ*G*_D–N_ for the three chicken brain spectrins. Further, visual inspection of the chevron curves in Fig. 2c reveals that the kinetic behavior of α0α1 is most similar to R15, a protein that folds by a nucleation mechanism with minimal landscape ruggedness.

β16 has behavior that is distinct from the previously studied spectrin domains. First, β16 is not observed to unfold in the presence of urea, requiring 4 M GdmCl to unfold half of the population. Indeed, β16β17 is twice as stable as α1, R15, R16, and R17, in terms of Δ*G*_D–N_ (Table 1). This remarkable stability has been previously observed [54] and may have a stabilizing effect on the neighboring tetramer domain, as has been the case for other spectrin domain pairs [55]. As seen in Fig. 2d, projection of the refolding limb of β16 suggests that, in the absence of denaturant, the protein folds as rapidly as α1 or R15, and projection of the unfolding limb of β16 reveals that the unfolding rate in water for the protein is much slower than anything previously seen for other members of the spectrin family.

As it is composed of two chains, the stability of the spectrin tetramer domain cannot be directly compared to R15, R16, and R17 since the equilibrium between associated and dissociated forms is concentration dependent. The second-order association/folding rate is also concentration dependent (*k*_+_ has units of M^−1^ s^−1^), and hence, *k*_+_ cannot be compared with folding rate constants for other spectrin domains (where *k*_f_ has units of s^−1^). However, the first-order disassociation/unfolding rate constant, *k*_−_, has units of s^−1^ and thus *can* be directly compared to the unfolding rate constants (*k*_u_, units of s^−1^) of the other spectrin domains. The non-covalent spectrin domain unfolds remarkably slowly, like the slow unfolding R17. With a *k*_−_ about three orders of magnitude lower than the unfolding rate of α1, the tetramerization domain is actually significantly more kinetically stable than its direct (covalent) neighbor. It is interesting to note that the dependence of ln(*k*_−_) on urea concentration (the dissociation/unfolding *m*-value, *m*_*k*−_) is very similar to the other spectrin domains (Fig. 3c and Table 1), suggesting that the transition state for unfolding of the tetramer domain is similarly expanded relative to the native state. We note that we have previously reported the dependence of the association rate constant, *k*_+_, on urea concentration (*m*_*k*+_). As expected, this has a weaker concentration dependence than for the other spectrin domains (Table 1) since α0 contains significant residual structure in its dissociated state, while the covalent spectrin domains have essentially disordered denatured states [56].

### Φ-Value analysis reveals transition state structure and suggests a folding mechanism

We have previously found the association of the spectrin tetramer to be consistent with a two-state mechanism for association/dissociation, without populated intermediates; rate constants estimated from kinetic data collected at various subunit concentrations were consistent with each other, and equilibrium and kinetic estimates of complex stability were in agreement [44]. Applying this model allows us to infer the structure of the transition state from either folding or unfolding kinetics. The vast majority of the mutants (with few exceptions, see below) had the same *m*_*k*−_ as the wild-type tetramer interaction, suggesting that these mutations did not alter the folding pathway. Some mutations effectively abolish binding, while others do not change the free energy of association sufficiently to allow us to determine a Φ-value with confidence (ΔΔ*G*_D–N_ < 0.7 kcal mol^−1^). Given this, we were able to determine Φ-values for 26 positions in the tetramer domain. A Φ-value of 1 indicates that the residue is in a region of the protein that is fully structured at the transition state; a Φ-value of 0 indicates that the region of the protein is as unstructured in the transition state as it is in the starting (denatured, unbound) state. As is standard practice, we classify Φ-values as high (largely structured), medium (partly structured), or low (largely unstructured), to give a qualitative picture of the structure of the transition state. Only three Φ-values (mutants Y94A, Y94L, and F97A) were non-classical, that is, significantly greater than 1 or less than 0.

**Fig. 5.**
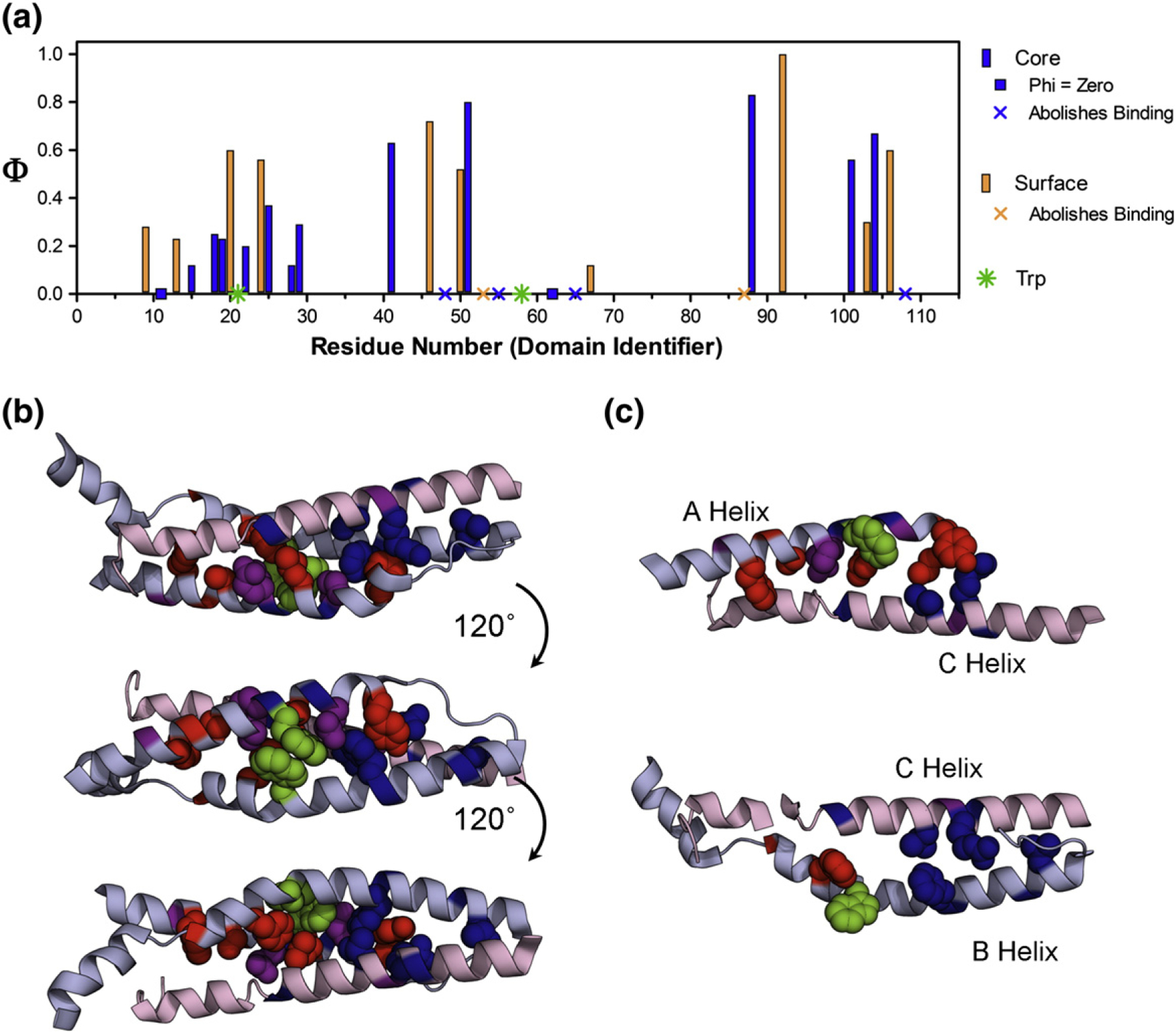
Results of Φ-value analysis. (a) The Φ-values form a coherent pattern when plotted against the sequence, covering a large range of both core (blue) and surface (orange) residues. The mutations that abolish binding are presented as symbols (x) in the appropriate color, and the two Trp residues, in Helices A and B, are shown as green symbols (✸). Peaks in the Φ-values for the A-helix (middle-to-C-terminal) and the B-helix (N-terminal-to-middle) indicate the most structured regions in β17 at the transition state. The Φ-values in the C-helix are more uniform. (b) Φ-values in the context of the structure of the spectrin tetramer are shown as spacefilling for core residues, while just the backbone is colored for surface residues (α1 and β16 have been removed for clarity). The Φ-values are divided as low (Φ < 0.25, red), medium (0.25 ≤ Φ < 0.5, purple), and high (Φ ≥ 0.5, blue). (c) Top, the A-helix and the C-helix are shown with their Φ-values in the absence of Helix B, which highlights the contact of high Φ-values in C-terminal Helix A with N-terminal Helix C at the transition state. The bottom structure shows only Helices B and C, where the contact between high Φ-values at the N-terminal end of Helix B with the C-terminal end of Helix C.

For the A-helix, we had good coverage: we determined ten Φ-values for nine buried residues (which monitor tertiary structure formation) and four surface Ala-to-Gly Φ-values (reporting on helix formation). The secondary structure Φ-values are higher than the tertiary Φ-values, and the Φ-values are higher in the middle/C-terminal end of the helix than at the N-terminal end (Fig. 5a).

In Helix B, four buried and two surface residue mutations in this helix abolished binding, while two others had mutations with ΔΔ*G*_D–N_ < 0.7 kcal mol^−1^. Thus, we could calculate only three surface and three buried Φ-values in this helix. Nevertheless, we can say that there is an indication of more structure formation at the N-terminal half of the B-helix (high Φ-values, 0.5–0.6 kcal mol^−1^) than for the C-terminal half, where Φ-values are close to 0.

**Fig. 6.**
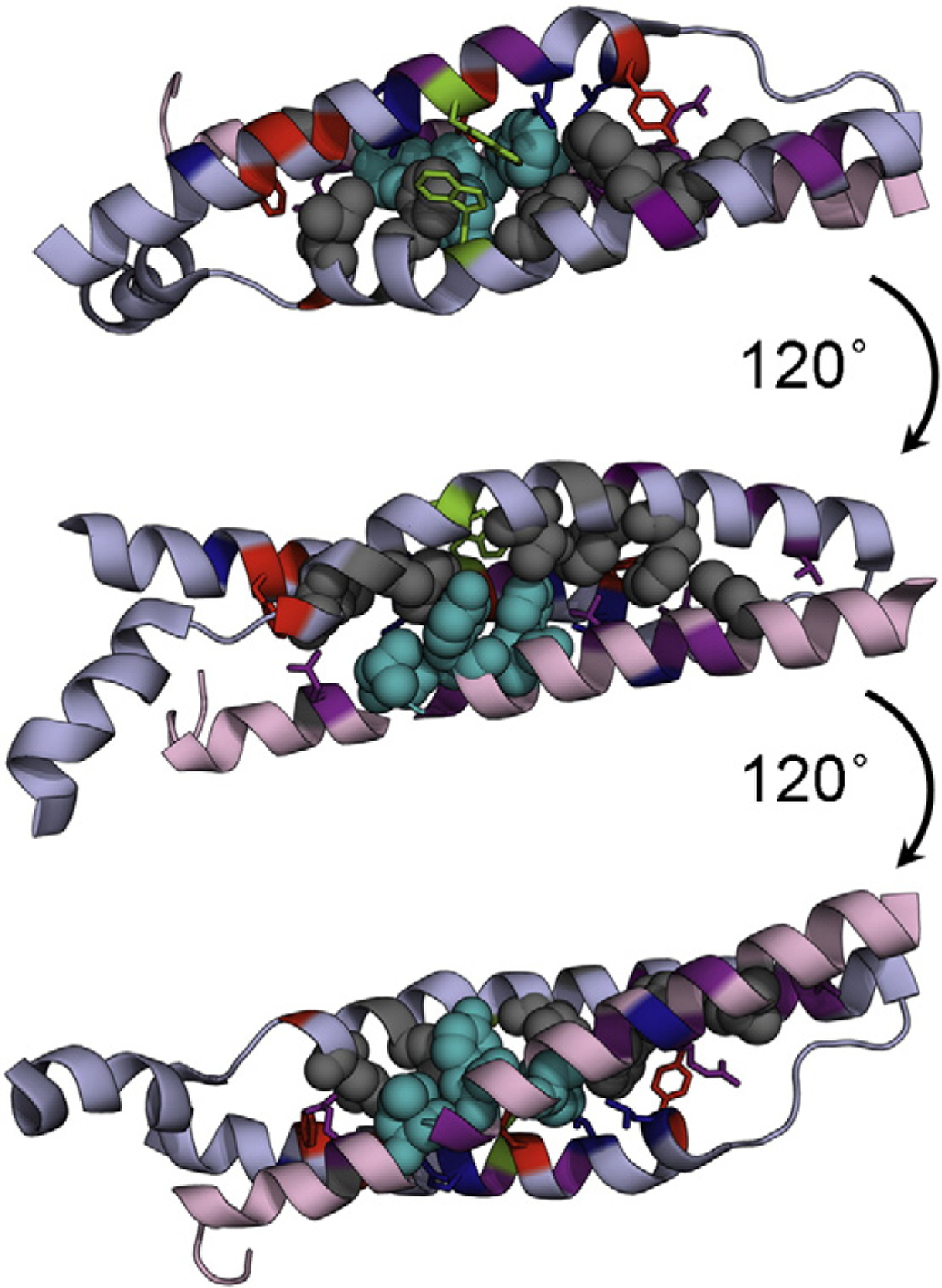
Mutations that abolish tetramer formation (gray) are primarily located within the B-helix, but two also occur in Helix C. Mutations that cause *m*_*k* −_ ≠ 1.2 M^−1^ or those that result in an unusual Φ-value are shown in cyan. Interestingly, all of these mutants occur in the C-helix, which is highly structured at the transition state for assembly/folding. Trp residues on the A- and B-helices are shown in green.

We determined six Φ-values in the C-helix, three buried and three solvent exposed. The tertiary Φ-values were, on average, higher than those in the Helices A and B and were consistently high along the helix. Helix C, which is apparently significantly structured in the unbound state, contained two residues whose mutation abolished binding, as well as two non-standard Φ-values (Fig. 6). We note that both R87A and L108A abolished formation of the tetramer domain and that physiological mutations leading to red blood cell deformation have been attributed to both of these residues, suggesting the critical importance of these residues in the mechanism of folding [57–60]. Buried residue Y94 has Φ-values of −2 (Y94A) and +4 (Y94L). F97A, in the next turn of helix, had a Φ-value of −0.3. L91A, for which we did not determine a Φ-value (low ΔΔ*G*_D–N_), had a slope that was inconsistent with the other mutants (Fig. S3). Each of these residues contacts the region of higher Φ-values from Helix A. Taken together, we propose that this interaction is important for the assembly of the tetramer domain and that mutation of this region of the preformed C-helix disrupts assembly.

**Fig. 7.**
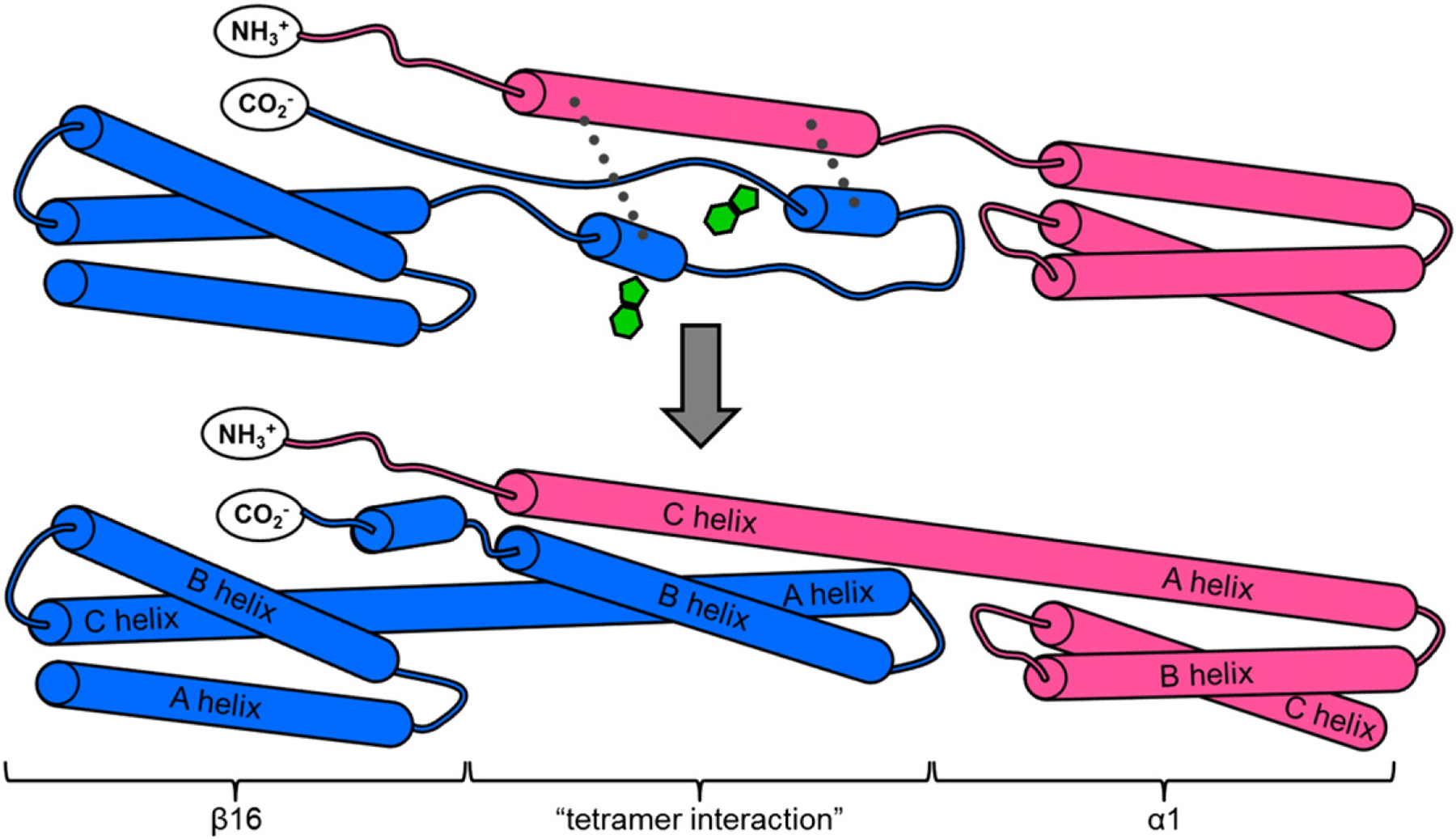
Cartoon depiction of proposed folding mechanism for the spectrin tetramer domain. β16β17 (blue) initially exists as a fully formed three-helix domain (β16) with a C-terminal tail with some transient helical structure. α0α1 (pink) has a partially structured α0 domain, followed by a fully structured α1. At the transition state, the middle-to-C-terminal end of the future A-helix comes into contact with the N-terminal end of the helical α0 domain, while the N-to-middle portion of the future B-helix contacts the C-terminal end of α0 (broken lines). Contact of β17 with the structured α0 helix stabilizes these nascent areas of secondary structure, ensuring the correct orientation and register for the helices, allowing folding to proceed efficiently. The two Trp residues (green), located on the A-helix and on the B-helix, are at the ends of the regions in these helices that pack onto C at the transition state.

We mapped the Φ-values onto the structure of the tetramer domain (Fig. 5b). It is clear that the two regions of higher Φ-values in Helix A and Helix B do not contact each other; thus, at the transition state, Helices A and B do not, apparently, dock. However, we see that the two elements of structure from β17 come into contact with the highly structured C-helix at the transition state. The middle-to-C-terminal part of the A-helix interacts with the N-terminal-to-middle part of Helix C, while the N-terminal end of the B-helix contacts the C-terminal end of the C-helix. Interestingly, three of the mutations in Helix B that disrupt structure formation are found in the N-terminal-to-middle region, which we propose binds Helix C during the transition state (Fig. 7).

These data are consistent with a mechanism whereby the preformed Helix C provides a template onto which Helices A and B dock (Fig. 7). There is some evidence that residual, possibly transient, helical structure in Helix A and/or B may exist, to which the C-helix may bind, thus stabilizing this residual structure. The docking of portions of Helices A and B onto Helix C establishes the correct topology and register of the helices, bringing the two Trp residues at the center of the core into contact (one from Helix A and one from Helix B), thus allowing folding to proceed efficiently.

## Conclusion

We have previously shown that spectrin domains can fold *via* nucleation condensation (R15) or by docking of partially preformed helices of A and B (R16 and R17). Here, we see yet another folding mechanism. The Φ-value analysis of the spectrin tetramer domain suggests a templating mechanism wherein transient elements of secondary structure in β17 dock onto the more highly structured C-helix. Our application of biophysical techniques and Φ-value analysis to the spectrin tetramer domain clearly demonstrates that enhanced understanding of IDP folding mechanisms can be achieved through the use of such methods, provided that care and stringent controls are used.

## Materials and methods

### Buffer and general conditions

All experiments were carried out in PBS [50 mM sodium phosphate and 150 mM NaCl (pH 7.04)]. The temperature for each experiment was held constant at 25 °C.

### Protein expression and purification

Wild-type α1, β16, α0α1, and β16β17 were expressed from synthetic genes (GenScript USA) in a modified pRSETA plasmid. All mutagenesis was carried out using a Stratagene QuikChange kit; identity of mutants was confirmed by DNA sequencing.

Protein expression and purification was carried out analogously to other spectrin domains [48]. The protein was expressed in *Escherichia coli* C41(DE3) cultured in 2× TY media at 37 °C in the presence of 0.1 mg ml^−1^ ampicillin. Cells were induced with 0.1 mg ml^–1^ IPTG, and the temperature was reduced to 25 °C once culture optical density at 600 nm reached 0.4–0.6 AU. After overnight incubation, cells were collected by centrifugation, then lysed by sonication and centrifuged again to separate the soluble protein from insoluble cell debris. The protein of interest was purified from the soluble fraction by affinity chromatography on Ni^+2^ agarose resin. Bound protein was released from the resin by thrombin cleavage and further purified with gel-filtration chromatography. Proteins were frozen in liquid N_2_ and stored for long periods at −80 °C. Once thawed for use, proteins were stored at 4 °C. Typical yield for α0α1 and β16β17 is 50 and 35 mg l^−1^ culture, respectively.

### Circular dichroism

Protein samples were prepared at 10 μM in PBS buffer. Spectra were collected in a 4-mm-pathlength or 1-mm-pathlength cuvette as follows: core residues, Chirascan CD Spectrometer (Applied Photophysics); Scan 250–208 nm, 1 nm bandwidth; surface residues and wild-type constructs, CD Spectropolarimeter (JASCO); Scan 260–200 nm, 0.5 nm bandwidth. Minor differences in spectra obtained from the two instruments were observed; however, no significant changes were observed between mutants.

### Stability of individual subunits

The stability of individual wild-type subunits was determined using urea- or GdmCl-induced denaturation. After excitation at 280 nm, intrinsic tryptophan fluorescence was collected from 300 to 450 nm on a PerkinElmer LS-55 fluorescence spectrophotometer. Protein concentration for these experiments was 5 μM; temperature was 25 °C.

### Kinetics of individual subunits

The folding and unfolding kinetics of the individual wild-type proteins were monitored by change in fluorescence, using an Applied Photophysics SX.20 stopped-flow spectrophotometer at 25 °C. Trp residues were excited with 280 nm light, and fluorescence data were collected above 320 nm. α0α1 and β16β17 were unfolded in urea and GdmCl, respectively, at a final protein concentration of 1 μM. Six traces were averaged for each concentration of denaturant and fitted to a single-exponential model, using KaleidaGraph (Synergy Software). In keeping with previous work using this instrument, we removed the first 2.5 ms prior to fitting to avoid mixing artifacts [47].

### Stability of the tetramer interaction

ITC experiments to determine the stability of the tetramer interaction were carried out on either an ITC200-Auto (core residues) or a VP-ITC (surface residues), both from MicroCal (GE). β16β17 at 25–50 μM in the cell was titrated with 15–20 injections to a total of 2–2.5 eq of α0α1, at 300 or 600 s intervals for core and surface residues, respectively. Each injection profile was integrated, and the resulting data were fitted with a one-site binding model in Origin 7 for MicroCal (OriginLab).

### Kinetics of the tetramer interaction

Folding and unfolding kinetics were monitored by intrinsic tryptophan fluorescence on either an Applied Photophysics stopped-flow spectrophotometer model SX.18 or SX.20 or a Biologic rapid mixing device. For both instruments, Trp residues were excited with 280 nm light and fluorescence was collected above 320 nm. A solution of pre-mixed α0α1 and β16β17, equilibrated 2 h, was mixed 1:1 with a urea solution. For all mutant unfolding data, the initial protein solution was 40 μM in each subunit, resulting in a final concentration in the cell of 20 μM in each subunit. Experiments were conducted at 25 °C, and traces were fitted as described in Results, using a fitting script in MATLAB (MathWorks). Unfolding rates were plotted against denaturant concentration and fitted globally using Igor (WaveMetrics).

## Supplementary Material

Supporting information

## Figures and Tables

**Table 1.** Equilibrium and kinetic data for α1, α0α1, β16, β16β17, and the tetramer domain

	*m*_D–N_ (kcal mol^−1^ M^−1^)	[Den]_50%_ (M)	Δ*G*_D–N_ (kcal mol^−1^)	$k_{\text{f}}^{{\text{H}}_{2}\text{O}}$ (s^−1^)	$k_{\text{u}}^{{\text{H}}_{2}\text{O}}\left(k_{-}^{{\text{H}}_{2}\text{O}}\right)$ (s^−1^)	$m_{k_{f}}$ (M^−1^)	$m_{k_{\text{u}}}$ (*m*_*k*−_) (M^−1^)
α1^a,c^	1.38 (±0.01)	3.47 (±0.01)	4.78 (±0.03)	—	—	—	—
α1 in α0α1^a^	1.73 (±0.03)	3.75 (±0.01)	6.49 (±0.03)	1500 (±400)	0.08 (±0.06)	1.3 (±0.1)	1.4 (±0.2)
β16	3.44 (±0.03)	4.19 (±0.01)	14.4 (±0.1)	5200 (±800)	3 × 10^−8^ (±2 × 10^−8^)	3.22 (±0.06)	2.7 (±0.1)
β16 in β16β17	3.3 (±0.1)	4.30 (±0.01)	14.2 (±0.1)	4000 (±3000)	1.7 × 10^−7^ (±1.5 × 10^−7^)	3.0 (±0.2)	2.7 (±0.1)
R15^a,d^	1.8 (±0.1)	3.7 (±0.1)	6.8 (±0.2)	25,000 (±8000)	2.0 (±0.5)	1.85 (±0.08)	1.00 (±0.03)
R16^a,d^	1.9 (±0.1)	3.6 (±0.1)	6.4 (±0.2)	136 (±4)	1.11 × 10^−2^ (±6 × 10^−4^)	2.08 (±0.02)	0.885 (±0.008)
R17^a,d^	2.0 (±0.1)	3.0 (±0.1)	6.0 (±0.2)	27.9 (±1.2)	5.7 × 10^−4^ (±4 × 10^−5^)	2.29 (±0.03)	1.46 (±0.01)
Wild-type tetramer^e^	—	—	—	—	2.5 × 10^−4^ (±3 × 10^−5^)	—	1.44 (±0.07)

a [Den]_50%_, *m*_D–N, $m_{k_{\text{f}}}$_, $m_{k_{\text{u}}}$, and *m*_*k*−_, reported in molar units of urea.

b Chevron not fitted.

c [Den]_50%_, *m*_D–N, $m_{k_{\text{f}}}$_, and $m_{k_{\text{u}}}$ reported in molar units of GdmCl.

d Data from Ref. [48].

e $k_{-}^{{\text{H}}_{2}\text{O}}$ and *m*_*k*−_ reported in lieu of $k_{\text{u}}^{{\text{H}}_{2}\text{O}}$ and $m_{k_{\text{u}}}$.

**Table 2.** Core mutants: thermodynamic and kinetic parameters and calculation of Φ-values

Domain identifier		Protein identifier	*K*_a_ (10^4^ M^−1^)		ΔΔ*G*_D–N_ (kcal mol^−1^)	*k*_−_ (10^−4^ M^−1^ s^−1^)	Φ^a^
*Wild type*			207 (±8)		—	4.7 (±0.3)	—
*β16β17*							
Helix A							
F11A		F2014A	0.6 (±0.6)		3.5 (±0.6)	2500 (±170)	−0.08 (±0.17)
F11L		F2014L	1.6 (±0.2)		2.93 (±0.09)	680 (±40)	−0.02 (±0.04)
A15G		A2018G	0.6 (±0.1)		3.51 (±0.11)	740 (±50)	0.14 (±0.03)
A18G		A2021G	13 (±3)		1.67 (±0.13)	35 (±3)	0.28 (±0.07)
E19A		E2022A	9.3 (±5)		1.9 (±0.3)	48 (±3)	0.25 (±0.13)
L22A		L2025A	0.6 (±0.1)		3.50 (±0.13)	470 (±30)	0.21 (±0.03)
Q25A		Q2028A	12 (±3)		1.80 (±0.14)	27 (±1)	0.39 (±0.06)
Y28A		Y2031A	18 (±2)		1.47 (±0.08)	41 (±3)	0.12 (±0.06)
Y28L		Y2031L	17 (±5)		1.50 (±0.19)	40 (±3)	0.15 (±0.11)
L29A		L2032A	29 (±2)		1.18 (±0.05)	19 (±2)	0.29 (±0.07)
Helix B							
V41A	V2044A			42 (±4)	0.96 (±0.06)	8.3 (±0.5)	0.65 (±0.07)
H48A^b^	H2051A			—	—	—	—
F51A^b^	F2054A			—	—	—	—
F51L	F2054G			18 (±3)	1.47 (±0.11)	6.2 (±0.4)	0.89 (±0.04)
T55A^b^	T2057A			—	—	—	—
F62A	F2065A			1.3 (±0.9)	3.0 (±0.4)	640 (±40)	0.3 (±0.14)
F62L	F2065L			10 (±2)	1.46 (±0.04)	48 (±3)	0.05 (±0.06)
L65A^b^	L2068A			—	—	—	—
*α0α1*							
Helix C							
Q88A^c^	Q29A			100 (±20)	0.42 (±0.1)	5.3 (±0.4)	—
L91A^c,d^	L32A			130 (±30)	0.27 (±0.14)	—	—
Y94A^c^	Y35A			67 (±2)	0.68 (±0.03)	148 (±10)	—
Y94L^c,d^	Y35L			440 (±50)	−0.45 (±0.07)	50 (±4)	
F97A	F38A			5.8 (±0.7)	2.14 (±0.07)	420 (±3)	−0.27 (±0.05)
F97L^e^	F38L			—	—	—	—
V101A	V42A			10 (±2)	1.80 (±0.13)	18 (±12)	0.6 (±0.2)
R104A	R45A			670 (±190)	−0.70 (±0.17)	3 (±3)	0.7 (±0.8)
L108A^b^	L49A			—	—	—	—

a Φ calculated using 〈*m*_*k*−_〉 = 1.2 M^−1^.

b Mutation abolished formation of the tetramer domain, as judged by ITC.

c ΔΔ*G*_D–N_ < 0.7 kcal mol^−1^; Φ not calculated.

d Slope of unfolding limb ≠ 1.2 M^−1^.

e Protein insoluble.

**Table 3.** Surface mutants: thermodynamic and kinetic parameters and calculation of Φ-values

Domain identifier	Protein identifier	*K*_a_ (10^4^ M^−1^)	ΔΔ*G*_D–N_ (kcal mol^−1^)	*k*_−_ (10^−4^ M^−1^ s^−1^)	Φ^a^
*Wild type*		207 (±8)	—	4.7 (±0.3)	—
*β16β17*					
C9A	C2012A	270 (±50)	—	3.7 (±0.3)	—
C9G	C2012G	47 (±1.2)	1.05 (±0.11)	1.3 (±0.9)	0.28 (±0.11)
R13A	R2016A	98 (±5)	—	7.8 (±0.5)	—
R13G	R2016G	7.1 (±0.4)	1.57 (±0.05)	60 (±4)	0.23 (±0.05)
S16A	S2019A	49 (±4)	—	6.2 (±0.5)	—
S16G	S2019G	36 (±5)	0.18 (±0.1)	11.6 (±0.8)	—
A20G	A2023G	53 (±8)	0.82 (±0.09)	7.9 (±0.6)	0.63 (±0.09)
A24G	A2027G	24.2 (±0.8)	1.29 (±0.03)	11.7 (±0.8)	0.58 (±0.05)
Helix B					
D39A^c^	D2042A	47 (±3)	—	6.4 (±0.5)	—
D39G^c^	D2042G	28 (±3)	0.3 (±0.07)	10.3 (±0.8)	—
K43A	K2046A	180 (±17)	—	4.4 (±0.3)	
K43G	K2046G	32.8 (±1.0)	1.02 (±0.06)	9.7 (±0.8)	0.54 (±0.07)
K46A^c^	K2049A	52 (±17)	—	12.7 (±1.0)	—
K46G^c^	K2049G	23.0 (±1.2)	0.5 (±0.2)	16.0 (±1.3)	—
A50G	A2053G	16.3 (±0.6)	1.52 (±0.04)	16.1 (±1.0)	0.52 (±0.04)
K53A	K2056A	150 (±13)	—	2.2 (±1.2)	—
K53G^b^	K2056G	—	—	—	—
S57A^c,e^	S2060A	150 (±15)	—	—	—
S57G^c^	S2060G	90 (±30)	0.32 (±0.18)	4 (±4)	—
A63G^b^	A2066G	—	—	—	—
K67A	K2070A	50 (±5)	—	12.0 (±1.0)	—
K67G	K2070G	14 (±2)	0.8 (±0.1)	37 (±3)	0.12 (±0.16)
*α0α1*					
Helix C					
E85A^c,e^	E26A	120 (±0.4)	—	—	—
E85G^c,e^	E26G	83.0 (±0.7)	0.221 (±0.010)	—	—
R87A^b^	R28A	—	—	—	—
R87G^d^	R28G	400 (±200)	—	4.1 (±0.9)	—
T92A	T33A	67 (±8)	—	6.0 (±1.1)	—
T92G	T33G	330 (±30)	−0.96 (±0.06)	7.8 (±1.5)	1.16 (±0.17)
Q95A^c,e^	Q36A	229 (±9)	—	—	—
Q95G^c,e^	Q36G	200 (±20)	0.08 (±0.02)	—	—
E99A^c^	E40A	90 (±9)	—	6.2 (±1.3)	—
E99G^c^	E40G	80 (±20)	0.04 (±0.16)	7.7 (±1.2)	—
E103A	E44A	81 (±9)	—	4.9 (±0.8)	—
E103G	E44G	22 (±3)	0.78 (±0.11)	12 (±2)	0.3 (±0.2)
Q106A	Q47A	130 (±14)	—	4.8 (±0.8)	—
Q106G	Q47G	20.0 (±1.8)	1.12 (±0.08)	10.2 (±1.7)	0.60 (±0.13)

a Φ calculated using *m*_*k*−_ = 1.2 M^−1^.

b Mutation abolished formation of the tetramer domain, as judged by ITC.

c ΔΔ*G*_D–N_ < 0.7 kcal mol^−1^; Φ not calculated.

d Slope of unfolding limb ≠ 1.2 M^−1^.

e Kinetic experiments not performed.

## Abbreviations used:

IDP: intrinsically disordered protein
GdmCl: guanidinium chloride
ITC: isothermal titration calorimetry
NIH: National Institutes of Health
