# Supplementary material for: Mechanism of Assembly of the Non-Covalent Spectrin Tetramerization Domain from Intrinsically Disordered Partners
Source: J Mol Biol. Author manuscript; Available in PMC 2022 May 9. (PMC9082959; doi:10.1016/j.jmb.2013.08.027)
Supplement: Supporting information [file NIHMS1803914-supplement-Supporting_information.pdf]

Supporting Information

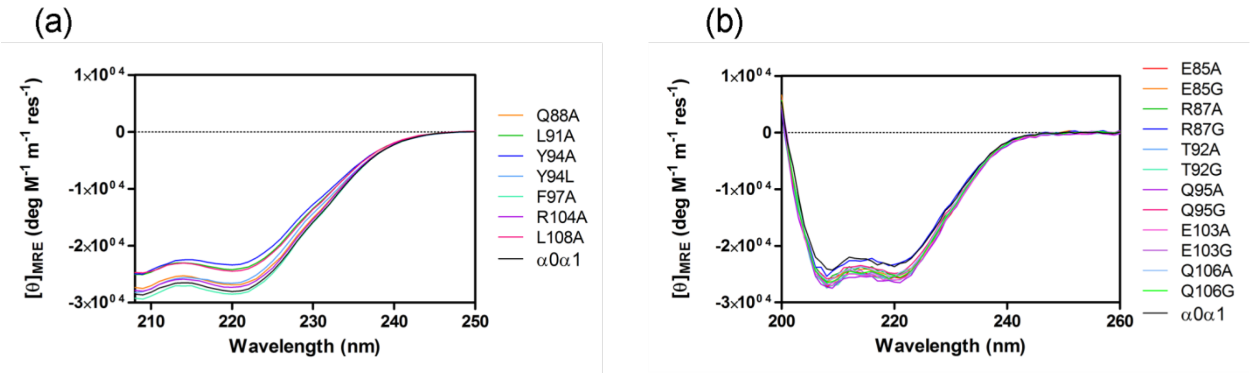

Fig. S1. Only three mutations reduce the residual helical structure in α0α1. (a) Core mutations L91A, Y94A, and L108A reduced the helicity of α0α1. Each of these mutants had interesting effects on the formation of the tetramer domain (see main text). (b) No surface mutations reduce MRE, but several increase the amount of helical structure in α0α1.

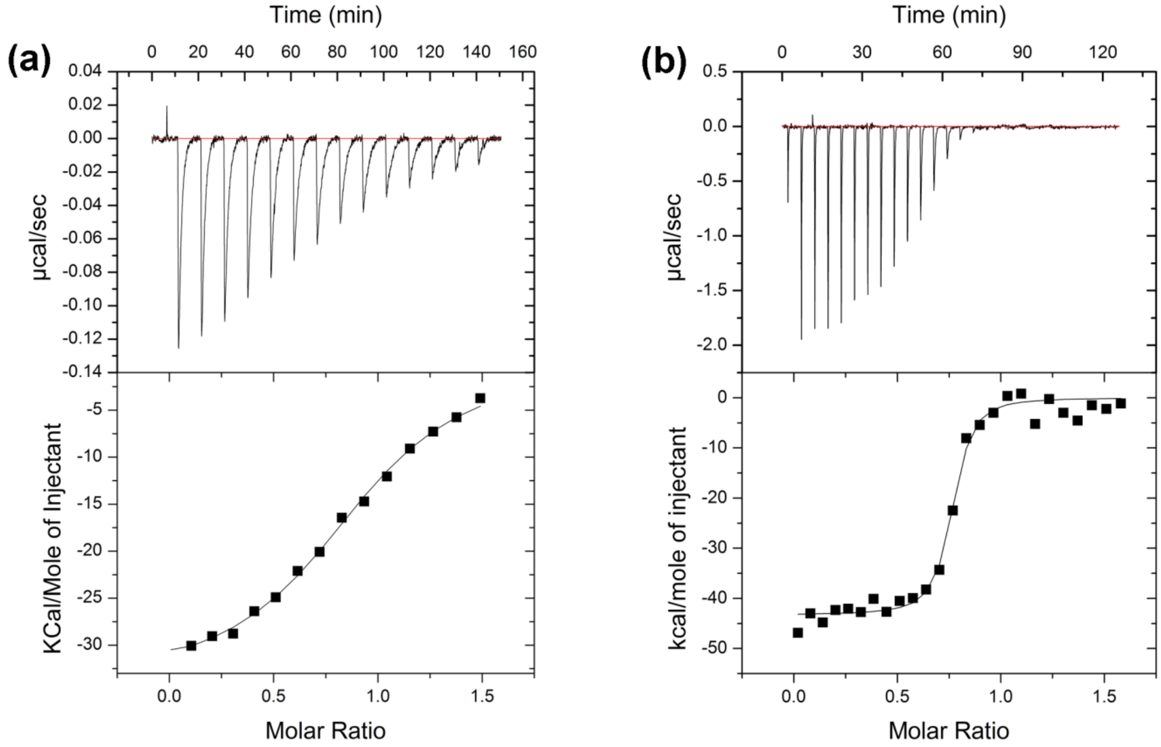

Fig. S2. Sample ITC experiments for weak binder K67A (a) and strong binder R104A (b). The increased affinity in R104A can be seen as an increase in the slope during the transition from unbound to bound complex. Additionally, the time required for the trace to return to baseline following each injection is higher for K67A, indicating slower association kinetics for this mutant versus R104A.

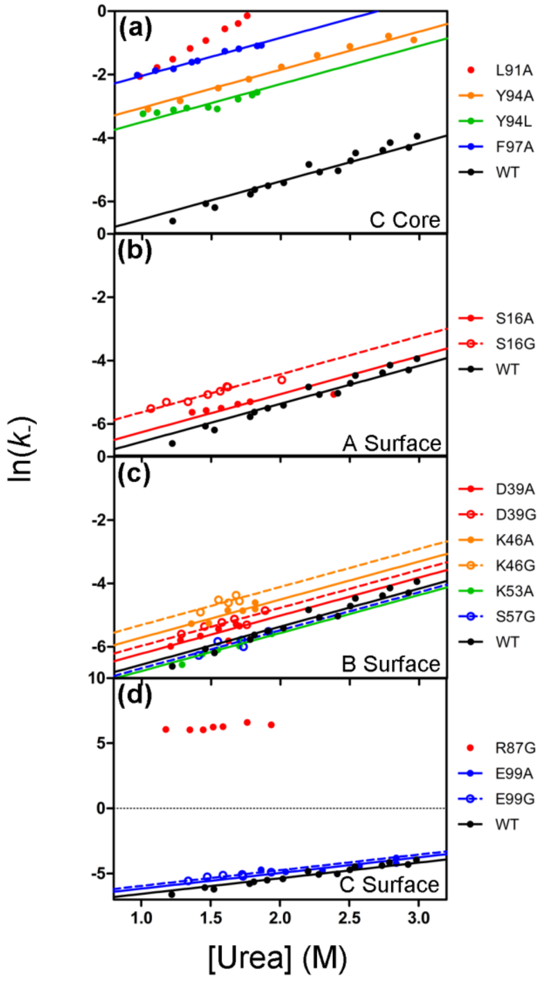

Fig. S3. Kinetic dissociation/unfolding limbs for which either a Φ-value was not calculated, or the Φ-value was non-classical ($\Phi > 0$ or $\Phi < 1$). (a) Mutants in C helix core: L91A has an unusual *m*-value, Y94A and Y94L have $\Delta\Delta G_{\mathrm{D\text{-}N}} < 0.7$ kcal mol^{-1}, F97A has a non-classical Φ-value. (b) Mutants in A helix surface: the S16A/G pair has $\Delta\Delta G_{\mathrm{D\text{-}N}} < 0.7$ kcal mol^{-1}. (c) Mutants in B helix surface: the D39A/G, K46A/G, S57G (versus S57A) pairs all have $\Delta\Delta G_{\mathrm{D\text{-}N}} <$ 0.7 kcal mol^{-1}. No Φ-value calculated for K53A/G, as K53G mutation abolishes binding. (d)

Mutants in C helix surface: R87G has an unusual *m*-value, and the E99A/G pair has $\Delta\Delta G_{D\text{-}N}$ < 0.7 kcal mol^{-1}.
